# Ethnic disparity in 21-hydroxylase gene mutations identified in Pakistani congenital adrenal hyperplasia patients

**DOI:** 10.1186/1472-6823-11-5

**Published:** 2011-02-18

**Authors:** Aysha H Khan, Muniba Aban, Jamal Raza, Naeem ul Haq, Abdul Jabbar, Tariq Moatter

**Affiliations:** 1 Department of Pathology and Microbiology, Aga Khan University, Stadium Road, Karachi, Pakistan; 2 Department of Pediatrics, Aga Khan University and National Institute of Child Health, Rafique Shaheed Road, Karachi, Pakistan; 3 Department of Medicine, Aga Khan University, Stadium Road, Karachi, Pakistan

## Abstract

**Background:**

Congenital adrenal hyperplasia (CAH) is a group of autosomal recessive disorders caused by defects in the steroid 21 hydroxylase gene (CYP21A2). We studied the spectrum of mutations in CYP21A2 gene in a multi-ethnic population in Pakistan to explore the genetics of CAH.

**Methods:**

A cross sectional study was conducted for the identification of mutations CYP21A2 and their phenotypic associations in CAH using ARMS-PCR assay.

**Results:**

Overall, 29 patients were analyzed for nine different mutations. The group consisted of two major forms of CAH including 17 salt wasters and 12 simple virilizers. There were 14 phenotypic males and 15 females representing all the major ethnic groups of Pakistan. Parental consanguinity was reported in 65% cases and was equally distributed in the major ethnic groups. Among 58 chromosomes analyzed, mutations were identified in 45 (78.6%) chromosomes. The most frequent mutation was I2 splice (27%) followed by Ile173Asn (26%), Arg 357 Trp (19%), Gln319stop, 16% and Leu308InsT (12%), whereas Val282Leu was not observed in this study. Homozygosity was seen in 44% and heterozygosity in 34% cases. I2 splice mutation was found to be associated with SW in the homozygous. The Ile173Asn mutation was identified in both SW and SV forms. Moreover, Arg357Trp manifested SW in compound heterozygous state.

**Conclusion:**

Our study showed that CAH exists in our population with ethnic difference in the prevalence of mutations examined.

## Background

Congenital adrenal hyperplasia (CAH; OMIM # 201910) is an autosomal recessive disorder, characterized by defects in any of the five-enzymes required to synthesize cortisol. In 95% of the cases, deficiency of 21-hydroxylase enzyme (21OH) was observed, which results in reduced synthesis of cortisol and aldosterone. Overproduction of adrenal androgens causes prenatal and postnatal virilization. CAH phenotypes vary with the degree of enzyme deficiency. Prenatal virilization in newborns females and salt wasting (SW-CAH) in both sexes, during the neonatal period can occur due to complete enzyme deficiency. On the other hand, partial enzyme deficiency leads to classic simple virilization form (SV-CAH) presenting with prenatal virilization in females and pseudo-precocious puberty in both males and females. Mild deficiency of 21OH causes non-classical disease (NC-CAH) with pseudo-precocious puberty, hirsutism, acne and reduced fertility at a later age [1].

Clinical diversity in CAH is primarily due to mutations in CYP21A2 gene, which encodes 21OH. CYP21A2 is located within the human leukocyte antigen class III region on the short arm of chromosome 6, adjacent to a highly homologous pseudo gene (CYP21A1P). Both are 3-4 kb long and each gene consists of 10 exons. Pseudo-gene has some deleterious mutations, which makes it non-functional. The majority of mutations in the steroid 21-hydroxylase gene have originated from this highly homologous adjacent pseudo-gene [1,2].

Analyses of different populations show that 95% of the CAH cases are caused by 9 specific point mutations and deletions in the CYP21A2 gene. Specific mutations produce a given degree of enzymatic compromise thus producing a clinical form of 21OH enzyme deficiency. In majority of patients, strong correlation between genotype and clinical disease presentation has been shown [3].

The data on CAH in Pakistan is very limited. In a previous study, we have reported CAH cases registered in our Hospital. Some of the features noted were delay in diagnosis, inappropriate gender assignment at birth, increase in the number of deaths in the family and high degree of consanguinity [4-6]. Moreover, as a result of late diagnosis, a large number of CAH patients in Pakistan develop genital malformations [6]. Therefore, this study was carried out to identify the spectrum of mutations in CYP21A2 gene, and to correlate genotype with the phenotype in a randomly selected cohort of CAH patients, which has been previously reported in our studies on CAH. The knowledge of the mutations in CYP21A2 gene will assist and promote prenatal diagnosis of CAH in the local population.

## Methods

### Study Subjects

This study was conducted at the Department of Pathology and Microbiology, The Aga Khan University and was approved by the Hospital Ethics Committee. Patients diagnosed with CAH were referred from Endocrine Clinics at The Aga Khan University Hospital and from the National Institute of Child Health, Karachi. CAH diagnosis was based on clinical presentation and hormonal investigation.

An independent clinical and biochemical evaluation of the patients was also performed at the time of inclusion, through a questionnaire. Informed consent for the study was obtained from patient's parents, or guardians. Patients were characterized into salt wasters (SW), simple virilizers (SV) and non-classical (NC) based on presentation and level of sodium, potassium and 17 hydroxy progesterone (17OHP).

CAH with SW was identified by the onset of failure to thrive, vomiting, hyperkalaemia, hyponatraemia, azotemia, metabolic acidosis, dehydration and shock requiring treatment with both mineralocorticoids and glucocorticoids. The SV form was identified by the presence of ambiguous genitalia in newborn females and penile enlargement, advanced bone age and pseudo precocious puberty in affected male children.

Blood samples were collected in tubes containing EDTA as anticoagulant. Genomic DNA was extracted from leukocytes using Promega Wizard genomic DNA purification kit (Promega, WI, USA). Purified DNA was screened for mutations in CYP21A2 gene using Amplified Refractory Mutation Specific Polymerase Chain Reaction (ARMS-PCR).

### Mutation Analysis

**Figure 1:**
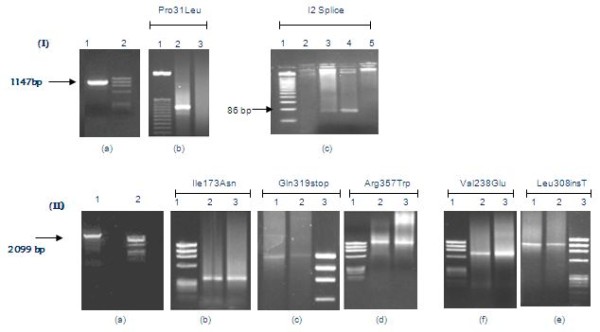
**Illustrates CYP21A2 gene mutations detected by allele-specific PCR (Ia) fragment A (Ib) Pro31Leu (Ic) I2splice (IIa) fragment B (IIb) Ileu173Asn (IIc) Gln319Stop, (IId) Arg357Trp (IIf) Val238Glu, (IIe) Leu308insT**. ϕX174 and 50 bp DNA markers were used for fragment size estimation.

ARMS-PCR was performed according to the protocol described by Wedell et al. [7]. Briefly, CYP21A2 gene was amplified in two overlapping fragments A and B, consisting of 1147 bp and 2099 bp respectively. Amplification of fragment A was carried in PCR buffer containing 0.5 μM primer P1 and P48, 1.5mM MgCl_2_, 0.2 mM dNTPs, 1 unit of *Taq *DNA polymerase and 250 ng patient's DNA. Cycling was performed at 94°C for 30 sec, 56°C for 35 sec, and 72°C for 1.30 min for 35 cycles. Fragment B was amplified using 0.5 μM of primers P4 and P55 and 250 ng DNA in 25 μl of High Fidelity PCR master mix (Roche Diagnostics, USA). Cycling program for fragment B was similar to fragment A, except for an increase in the extension time to 2.30 min. In the second round PCR, fragment A was amplified with primers specific for mutations at nucleotide positions 659 (I2 splice) and 92 (Pro 31leu) and fragment B was assessed for mutations 1004 (Ile173Asn), 1388 (Val238Glu), 1688 (Val282Leu), 1768 (Leu308InsT), 1999 (Gln319stop) and 2113 (Arg357Trp). Nucleotide positions refer to the CYP21A2 gene accession number M12792. Subsequent to PCR, amplified products were separated on 1% agarose gel and visualized by ethidium bromide staining (figure 1).

## Results

Table 1 summarizes clinical and biochemical details and genotype status of CYP21A2 in our study participants. At the time of diagnosis, mean age of the patients was 2.2 years. There were 14 phenotypic males and 15 females (female to male ratio of 1.07). Among females, 13 had features of prenatal virilization at birth and those were initially considered to be male before the final diagnosis. In 5 cases, male sex was preserved and they were raised as male. One of the patients diagnosed for CAH at the age of 47 years, turned out to be a case of late diagnosis, although this patient had history of virilizing symptoms since birth. According to the clinical and laboratory investigations, 17 patients were salt wasters, whereas 12 patients were simple virilizers. Median 17 OHP levels in salt wasters and in simple virilizers were 140 ng/ml and 60 ng/ml respectively. 17 OHP levels were consistently higher in salt wasters as compared to simple virilizers but this difference was not statistically significant. However, hyponatremia and hyperkalemia was significantly more marked in salt wasters as compared to simple virilizers (p < 0.002).

**Table 1:** Clinical, Biochemical & Genotype Status of CAH Patients

ID	Sex	Ethnicity	Genotype	Phenotype	Na*	K*	17 OHP
1	M	-	I2 splice/I2 splice	SW	122	8.6	273.2
2	M	-	I2 splice/-	SV	140	3.8	0.1
3	M	Urdu Speaking	Arg357Trp/-	SV	137	4.4	57
4	F	Urdu speaking	I2 splice/I2 splice	SW	124	6	>400
5	M	Afghani	ND	SV	141	5.6	51
6	F	Urdu speaking	I2 splice/-	SV	130	3.4	59.4
7	F	Urdu speaking	I2 splice/I2 splice	SW	140	6.6	201
8	F	Sindhi	Pro31Leu/Ile173Asn/Arg357Trp/Gln319stop/Leu308insT/Val238Glu	SW	125	7.9	400
9	F	Punjabi	Arg357Trp/Gln319stop/Leu3081nsT/	SW	136	5.2	1.4
10	F	Sindhi	Ile173Asn/Ile173Asn	SV	139	4.3	226.4
11	M	Punjabi	Pro31Leu/Pro31Leu	SW	98	7	185.8
12	M	Pathan	Ile173Asn/Arg357Trp/Gln319stop/Leu3081nsT	SW	115	7.9	31.8
13	M	Sindhi	Arg357Trp/Gln319sto/Val238Glu	SW	104	7.8	>400
14	F	Punjabi	Ile173Asn/Ile173Asn/Arg357Trp/Gln319stop/Leu308insT/Val238Glu	SV	-	-	60.8
15	F	Punjabi	ND	SV	143	3.6	150
16	M	Pathan	Arg357Trp/Gln319stop/Leu3081nsT	SW			9.5
17	M	Afghani	Ile173Asn/Ile173Asn/Arg357Trp/Leu3081nsT	SW	147	5.8	125
18	F	Afghani	Ile173Asn/Ile173Asn/Arg357Trp/Gln319stop/Leu308insT/Val238Glu	SV	131	4.6	48.2
19	M	Urdu speaking	Ile173Asn/Ile173Asn/Arg357Trp/Gln319stop	SV	141	4.6	>400
20	M	Sindhi	Pro31Leu/Arg357Trp/Val238Glu/Gln319stop	SV	134	5.1	92
21	M	Pathan	I2 splice/-	SV	139	5.2	9.2
22	F	Punjabi	ND	SV	139	4.4	20
23	F	Pathan	I2 splice/I2 splice	SW	128	5.3	166.4
24	M	Urdu Speaking	I2 splice/-	SW	134	4.3	77.8
25	F	Urdu speaking	I2 splice/I2 splice	SW	128	5.2	>20
26	M	Afghani	Pro31Leu/-	SW	115	5.7	97.6
27	F	Punjabi	Ile173Asn/Ile173Asn	SW	120	5	>400
28	F	Urdu Speaking	I2 splice/I2 splice	SW	-	-	109.8
29	F	Sindhi	Ile173Asn/-	SW	122	6	21.4

Na^+:^: sodium; K^+^: potassium; 17OHP: 17 hydroxyprogestron; SW: salt wasters; SV: simple virilizers

**Figure 2:**
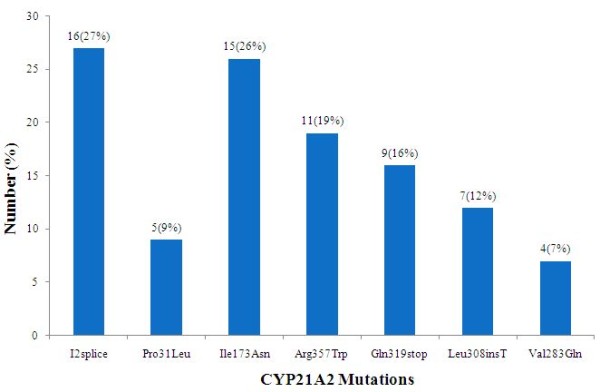
**Distribution of common point mutations in CAH patients (n = 29)**.

The patients represented most of the major ethnic groups of Pakistan (Urdu speaking 27%, Punjabi 21%, Sindhi 17%, Pathans 14% and Afghanis 14%). Urdu speaking and Afghanis comprised of the people who migrated from India and Afghanistan at the time of sub-continent partition and during the Afghanistan war. While Sindhi, Punjabi and Pathans are the local inhabitants. Ethnicity was not known in 6.8% of the cases. Parental consanguinity was present in 65% cases and was seen in all the ethnic groups examined. In 6 families more than 1 child was affected. Table 1, illustrates the distribution of eight mutations common to our patients' population. Evaluation of 58 chromosomes revealed mutations in 45 (78%) chromosomes. The most frequently occurring mutation was I2 splice (27%) followed by Ile173Asn (26%), Arg 357 Trp (19%), Gln319stop 16% and Leu308InsT (12%), whereas Val282Leu was not detected in this study (figure 2). Among Urdu speaking patients, (17%) were SW and (10%) were SV, but overall 62% of them had I2 splice mutation. High frequency (50%) of I2 mutation was also encountered in Pathans. On the contrary, none of the patients from Sind, Punjab and Afghanistan showed I2 mutation. When compared among ethnic groups, Ile173Asn was most frequently encountered in Sindhis (75%) and Afghanis (50%). High level of compound heterozygosity was also noted in Sindhis (60%), Pathans (50%) and Afghanis (50%). Out of 29 patients, 19 showed mutations in both the alleles, whereas seven patients demonstrated mutation in one allele. In addition, three patients were normal for all eight mutations evaluated. Homozygosity was seen in 13 patients (44%). Out of these patients, 46% were homozygous for both I2 splice and Ile173Asn mutations and 7.6% were homozygous for Pro31Leu. Furthermore, heterozygosity was noted in 10 (34%) patients. Relationship between genotypes and the phenotypes were studied by comparing disease manifestation in patients with different combination of mutations. I2 splice mutation was found to be associated with SW in homozygous state, while presence of I2 splice mutation in one allele was found to be associated with the SV form of the disease. Additionally, Ile173Asn mutation was observed in both SW and SV forms and Arg357Trp mutation was manifested in SW and SV in the compound heterozygous state.

## Discussion

The health issues of gender ambiguity are not well studied in the Pakistan population. According to the local literature, children with CAH are usually brought to medical attention late in life [4,5]. It is also evident from the present study that the median chronological age at the time of diagnosis was comparatively high and only 41% cases were diagnosed during the newborn period [4,8]. The age at diagnosis in our study is much higher, in comparison to the countries that have an established newborn screening program. However, the benefits of screening for any disease in a country are dependent on the existing healthcare system. The management of genetic disorders has not received its due share in Pakistan, additionally; its healthcare system generally does not address complex issues commonly seen in genetic disorders including CAH patients [9,10]. Furthermore, the matter is exacerbated by the high degree of parental consanguinity seen in the local population (65.5%). Therefore, there is a need to set up an infrastructure with basic research and medical facilities with genetic testing and counseling for CAH patients so that better translational research concepts and intervention strategies are designed to help consanguineous couples for making informed and intelligent reproductive decisions.

Another important issue that relates to CAH in Pakistan is the large ethnic diversity. Main ethnic groups include Punjabis (44), Pathans (15%), Sindhis (14%), Seraikis (11%), Muhajirs (8%), Balochis (4%) and few others. In addition, Afghan refugees (registered in Pakistan as *Afghan Citizens*) from neighboring Afghanistan are also present [11]. Cultural norms of all ethnicities in Pakistan place a higher value on beneficence and non-malfeasance relative to autonomy. They have a long tradition of making family-centered health care decisions. In this collective decision making process, relatives receive information about patient's diagnosis and prognosis and make treatment choices, often without the patient's input especially if the patient is a female. Illnesses like CAH are considered a family matter rather than an individual occurrence. Physicians are challenged by tremendous diversity within a specific ethnic group that influences the family and patient response to medical issues of CAH and depends on traditional views of gender roles, child bearing practices, views of marriage and relationships, common religious and spiritual beliefs. Females born with ambiguous genitalia continue to suffer from the consequences of genital ambiguity, even if the diagnosis was made at birth and in most of the cases they often do not receive treatment. In our study, wrong gender assignment to genotypic females is noted and in some of them male sex was maintained due to late presentation and development of male gender identity at diagnosis (5 cases, 17.2%). It is a result of lack of awareness, failure to diagnosis, false social beliefs, religious dogmas, pressure from relatives and society in this part of the world. Majority of the CAH causing mutations are sequences that are normally present in the CYP21P gene, which can cause CYP21A2 gene inactivation if transmitted to the functional gene by the process of gene conversion. This accounts to approximately 95% of all mutated CYP21A2 alleles. The remaining 5% of the mutations are population specific and are not derived from the pseudo gene. Analysis of the CYP21A2 gene presents a challenge because of the presence of this highly homologous CYP21P. To overcome this obstacle, several selective CYP21A2 gene amplification and mutation detection procedures are developed [12-17]. In this study we have adopted a strategy to detect eight most common CYP21A2 point mutations based on hybridization with allele specific primers that can discriminate between mutation and wild type sequences, whereas pseudo gene was excluded by selective amplification [7]. Our results showed that seven CYP21A2 gene mutations were detected in 78% (45/58) of CAH patients. As shown in table 2, frequency of mutant alleles in Pakistani patients was comparable to other parts of the world including Iran and Turkey [18,19]. Among all the known monogenic disorders, prenatal treatment for CAH is the most effective. Therefore, there is a need to establish a rapid, convenient prenatal screening method for diagnosis at least in families with known CAH patients [6].

**Table 2:** Comparison of CAH alleles in Different Populations

Country	Total alleles	I2 Splice	Ile173Asn	Arg 357Trp	Pro31Leu	Gln319stop	Val283Glu	Reference
Pakistan	58	27	26	19	9	16	7	This study
Iran	100	28	9	0	0	0	3	18
Turkey	31	22	11.4	0	0	0	0	19
India	46	27.2	31.8	0	2.2	22.7	0	20

In this study, 29 patients of CAH due to 21 OH deficiency were evaluated. Seventeen (59%) of them exhibited the SW features and 12 were confirmed as SV (41%). The frequencies for SW and SV forms of the disease were different from published studies. For example, in an Iranian study, SW constituted 70% of the total CAH cases, whereas 30% were SV [18]. On the other hand, the spectrum and frequency of mutations including I2 splice, Ile173Asn and Gln319stop presented in study were very similar to those reported in the neighboring Indian population [20]. Our PCR amplification failed in three cases. In two of them, fragment B was not amplified, which could be due to the presence of homozygous deletions/conversions extending to exons 3, 4 and 5. Currently, it was not possible for us to perform large deletions by methods like MLPA, because of limited resources. However, we like to include it in future studies. In one case neither of the fragments amplified, which may be due to homozygous gene deletion of CYP21A2 or to transfer into CYP21A2 of pseudogene sequences that include the 8 bp deletion in exon 3 [7]. Analysis of homozygous individuals in our study showed that I2 mutation in the homozygous state was associated with SW forms of the disease, whereas Ile173Asn was associated with both SV and SW forms of the disease. All these cases had markedly elevated 17OHP levels. Compound heterozygosity was noted in both SV and SW forms of the disease. In our study, compound heterozygosity was seen more frequently in Sindhis, Pathans and Afghanis in comparison to Punjabis or Urdu speaking communities. Moreover, compound heterozygous genotypes were seen in SV, whereas a higher frequency of homozygous genotypes was observed in SW. The high rate of homozygosity in SW may be due to high degree of parental consanguinity seen in our patients (70%). It is also possible that the cases reported as homozygous in our study may have been heterozygous, because we did not examine the allele frequency for large deletions and conversions [18]. It is our experience that the process of disclosure of all aspects of CAH and its clinical care will require a collaborative effort. For optimal care, experienced multidisciplinary teams in tertiary care centers should openly communicate with patients and their families. Thus allowing patients to participate in making their own decisions, while respecting their confidentiality and family's concerns.

## Conclusion

It is evident from our study that CAH has clear presence in Pakistan, which is reflected in all major ethnic groups. Extensive parental consanguinity and multi ethnic makeup are major factors leading to its widespread prevalence. Therefore, there is a prompt necessity to set up national and regional neonatal screening programs while including CYP21A2 genotyping in the diagnostic component to address this serious health issue.

## Competing interests

The authors declare that they have no competing interests.

### Financial Competing interests

In the past five years we have not received reimbursements, fees, funding, or salary from an organization that may in any way gain or lose financially from the publication of this manuscript

We do not hold any stocks or shares in an organization that may in any way gain or lose financially from the publication of this manuscript.

We are not having or are currently applying for any patents relating to the content of the manuscript. We have not received reimbursements, fees, funding, or salary from an organization that holds or has applied for patents relating to the content of the manuscript

We do you have any other financial competing interests

### Non-financial competing interests

There aren't any non-financial competing interests (political, personal, religious, academic, ideological, intellectual, commercial or any other) to declare in relation to this manuscript

## Authors' contributions

**AHK **conceived the study, and developed its design; contributed in patients' assessment, sampling and biochemical testing. Also, participated in the analysis of molecular studies and patients' counseling and manuscript writing. **MA **performed molecular testing, data collection and participated in results analysis and manuscript writing. **JR **contributed in patients' selection and follow-ups. **NH **contributed in patients' selection and follow-ups. **AJ **contributed in patients' selection and follow-ups. **TM **was involved in the study design, methods validation, data analysis, manuscript writing and review. All authors read and approved the final manuscript

## Pre-publication history

The pre-publication history for this paper can be accessed here:

http://www.biomedcentral.com/1472-6823/11/5/prepub

## References

[B1] Speiser PW White PC Congenital adrenal hyperplasia N Engl J Med 2003 349 776 10.1056/NEJMra021561 12930931

[B2] Levine LS Congenital adrenal hyperplasia Pediatr Rev 2000 21 159 170 10.1542/pir.21-5-159 10790482

[B3] Wedell A Molecular genetics of congenital adrenal hyperplasia (21-hydroxylase deficiency): implications for diagnosis, prognosis and treatment Acta Paediatr 1998 87 159 164 10.1080/08035259850157598 9512201

[B4] Bhanji R Khan AH Balouch IL Sabir S Nazir Z Billoo AG Profile of Children with Congenital Adrenal Hyperplasia-A Hospital Study J Pak Med Assoc 2004 54 509 512 15552284

[B5] Khan AH Nasir MI Moatter T Characterization of pathogenic mutations in 21-hydroxylase gene of Pakistani patients with congenital adrenal hyperplasia -a preliminary report J Pak Med Assoc 2002 52 287 291 12481658

[B6] Khan AH Aban M Hassan RU Haq NU Raza J Jabbar A Moatter T Classic virilizing congenital adrenal hyperplasia presenting late: case series from Pakistan J Pak Med Assoc 2009 59 643 646 19750867

[B7] Wedell A Luthman H Steroid 21-Hydroxylase deficiency: two additional mutations in salt wasting disease and rapid screening of disease causing mutations Hum Mol Genet 1993 2 499 504 10.1093/hmg/2.5.499 8518786

[B8] Thilen A Nordenstrom A Hagenfeldt UVD Guthenberg C Larsson A Benefits of Neonatal Screening for Congenital Adrenal Hyperplasia (21-hydroxylase deficiency) in Sweden Paediatrics 1998 101 E11 10.1542/peds.101.4.e11 9521977

[B9] Pakistan faces genetic disasters http://english.ohmynews.com/articleview/article_view.asp?menu=f10600&no=321444&rel_no=1

[B10] Pakistan lack of healthcare access http://www.irinnews.org/report.aspx?ReportId=74825

[B11] Demographics of Pakistan http://www.wikipedia.org/wiki/Demographics_of_Pakistan

[B12] Wilson RC Nimkarn S Dumic M Obeid J Azar MR Najmabadi M Saffari F New MI Ethnic specific distribution of mutations in 716 patients with congenital adrenal hyperplasia owing to 21 hydroxylase deficiency Mol Genet Metab 2007 90 414 421 10.1016/j.ymgme.2006.12.005 17275379 PMC1885892

[B13] Wedell A Astrid T Ritzen EM Mutational spectrum of 21-hydroxylase gene in Sweden: Implications for genetic diagnosis and association with disease manifestation J Clin Endocrinol Metab 1994 78 1145 1151 10.1210/jc.78.5.1145 8175971

[B14] Lobato MN Ordonez SM Tusie-Luna MT Mesequer A Mutation analysis in-patients with congenital adrenal hyperplasia in the Spanish population: identification of putative novel steroid 21-hydroxylase deficiency alleles associated with classic form of the disease Hum. Hered 1999 49 169 175 10.1159/000022866 10364682

[B15] Bachega TAAS Billerbeck AEC Madureira G Marcondes JAM Longui CA Leite MV Arnhold IJP Mendonca BB Molecular genotyping in Brazilian patients with the classical and non-classical forms of 21-hydroxylase deficiency J Clin Endocrinol Metab 1998 83 4416 4419 10.1210/jc.83.12.4416 9851787

[B16] Asanuma A Ohura T Ogawa E sato S Igarashi Y Matsubara Y Iinuma K Molecular analysis of Japanese patients with steroid 21-hydroxylase deficiency J Hum Genet 1999 44 312 317 10.1007/s100380050167 10496074

[B17] Bobba A Merra E Giannattasio S Lolascon A Monno F Di Maio S 21-hydroxylase deficiency in Italy: a distinct distribution pattern of CYP21 mutations in a sample from Southern Italy J Med Genet 1999 36 648 650 10465121 PMC1762977

[B18] Ramazani A Kahrizi K Razaghiazar M Mahdieh N Koppens P The frequency of Eight Common Point Mutations in CYP21 Gene in Iranian Patients with Congenital Adrenal Hyperplasia Iran Biomed J 2008 12 49 53 18392095

[B19] Tukei T Uyguner Wei Jo Yukset AM Saka N Song DX Kayseridi H Bas F Gunoz H Wilson RC New MI Wollink B A novel semiquantitative polymerase chain reaction/enzyme digestion-base method for detection of large scale deletions/conversions of the CYP21 gene to mutations screening in Turkish families with 21 hydroxylase deficiency J Clin Endocrinol Metab 2003 88 5893 5897 10.1210/jc.2003-030813 14671187

[B20] Mathur R Menon PS Kabra M Goyal RK Verma IC Molecular characterization of mutations in Indian children with congenital adrenal hyperplasia due to steroid 21- hydroxylase deficiency J Pediatr Endocrinol Metab 2001 14 27 35 11220701 10.1515/jpem.2001.14.1.27

